# Glue ablation therapy for patients with great saphenous vein varicosities: A randomized, parallel-controlled, multicenter, non-inferiority trial

**DOI:** 10.1515/jtim-2025-0054

**Published:** 2025-12-12

**Authors:** Kangkang Zhi, Sili Zou, Yi Hong, Lianrui Guo, Yixia Qi, Lei Zhang, Junlu Peng, Bo Ye, Guofu Zheng, Bin Hao, Huimin Xu, Bin Chen, Yifeng Pan, Yuefeng Zhu, Jinjin Wu, Xiaojin Huang, Shenci Wen, Xiang Wang, Lefeng Qu

**Affiliations:** Vascular Surgery Department, Changzheng Hospital, Naval Medical University, Shanghai, China; Vascular Surgery Department, Shanghai East Hospital, Tongji University, Shanghai, China; Vascular Surgery Department, Xuanwu Hospital, Capital Medical University, Beijing, China; Vascular Surgery Department, the First Hospital of Hebei Medical University, Hebei Province, China; Ganzhou People's Hospital, Jiangxi Province, China; Shanxi Dayi Hospital, Shanxi Province, China; The Second Affiliated Hospital, Zhejiang University, Zhejiang Province, China; The Fourth Affiliated Hospital, Zhejiang University, Zhejiang Province, China; Zhongshan Hospital, Xiamen University, Fujian Province, China

**Keywords:** glue ablation, radiofrequency ablation, great saphenous vein varicosities

## Abstract

**Background:**

Treatment for varicose veins has transitioned from invasive surgical interventions to minimally invasive, targeted, and personalized procedures. Limited data exist on the longterm outcomes of glue ablation (GA) as a new minimally invasive treatment. This study was conducted to evaluate the long-term safety and efficacy of GA for varicose veins.

**Methods:**

Data were collected in a multicenter, prospective registry. Patients were randomly allocated to the experimental group (GA) or the control group (radiofrequency ablation [RFA]). The follow-up assessments were conducted at 7 days, 30 days, 3 months, 6 months, and 12 months after surgery. The non-inferiority of GA to RFA was assessed based on the primary outcome of the rate of complete great saphenous vein (GSV) closure at 3 months. Additional outcomes included the rate of complete GSV closure at 12 months, procedural duration, pain score, ecchymosis, and preoperative and postoperative Venous Clinical Severity Score and Aberdeen Varicose Vein Questionnaire Score.

**Results:**

Overall, 177 patients were treated (experimental group, *n* = 89; control group, *n* = 88). The mean age was 56.25 ± 12.23 years, and 61.58% were female. The mean target vessel diameter was 7.94 ± 2.09 mm, with a maximum diameter of 12 mm. The GA group did not experience any device-related adverse complications. Tumescent fluid was utilized in the RFA group but not in the GA group. GA was statistically non-inferior to RFA (lower boundary of the 95% confidence interval [CI] for the absolute difference in the mean rate of 3-month complete GSV closure did not reach the non-inferiority margin of -10%) (absolute difference -0.03%; one-sided 95% CI -3.19%; *P* < 0.001). The GSV closure rate at 12 months was higher in the GA group than in the RFA group (absolute difference 7.04%; one-sided 95% CI 1.09%). The GA group exhibited a significantly longer surgical duration than the RFA group (*P* = 0.031). The comparisons of other secondary endpoints between the groups did not yield any significant findings.

**Conclusion:**

The 3-month rate of complete GSV closure with GA was non-inferior to that with RFA in the treatment of varicosity, but the surgical duration was longer. The efficacy of GA therapy still requires validation through large-scale clinical trials with long-term follow-up periods.

## Introduction

Disruption of the normal function of the venous system leads to retrograde flow, otherwise known as venous incompetence.^[1,2]^ Chronic venous insufficiency, a type of long-standing venous incompetence, affects approximately one-third of the adult population,^[1]^ with saphenofemoral or great saphenous vein (GSV) valvular incompetence accounting for 60%–70% of cases.^[3]^ Patients with this condition may experience minor symptoms, such as aching, edema, and poor appearance.

The traditional treatment for GSV incompetence is open surgery, namely high ligation and stripping (HL/S). The postoperative recovery period of HL/ S typically spans from 2 to 3 weeks, with an overall complication rate of 17%–20%.^[4,5]^ In the past two decades, there has been a rise in minimally invasive procedures for the treatment of GSV incompetence, encompassing thermal and non-thermal therapies.^[1,6]^ Thermal therapies rely on the utilization of heat energy to induce damage to the vein wall, resulting in occlusion and fibrosis, while non-thermal therapies predominantly utilize a chemical sclerosant.

Radiofrequency ablation (RFA) is a safe and effective treatment for chronic venous diseases with a high longterm target vein closure rate.^[7,8]^ Moreover, glue ablation (GA) has recently emerged as a novel therapeutic approach that can induce inflammatory and chemical damage to the vein wall. However, the existing evidence is inadequate to substantiate the long-term clinical efficacy of GA. In this study, we evaluated the efficacy and safety of GA compared with RFA over a duration of 12 months.

## Methods

This study was a prospective, multicenter, randomized, parallel-controlled, non-inferiority clinical trial. The GA system (Vengenius™ Closure System [VG005], Shanghai Yisimiao Medical Equipment Co. Ltd.) was compared with the RFA system (ClosureRFG™ [RFG3]; ClosureFast™ [CF7-7-60; CF7-7-100], Medtronic Inc.) . Adverse events, procedural complications, and changes in laboratory tests before and after the procedure were recorded from the time of informed consent acquisition until the completion of follow-up.

The inclusion criteria were patients (1) aged between 18 and 75 years, (2) who presented with a clinical diagnosis of GSV varicosities, and (3) who exhibited a Clinical–Etiology–Anatomy–Pathophysiology grade ranging from C2 to C4b. All participants volunteered to participate and provided written informed consent.

Participants were excluded if they (1) required daily administration of anesthesia or non-steroidal anti-inflammatory drugs for pain management associated with varicose veins; had (2) severe liver or renal dysfunction (alanine transaminase > 3-times the upper limit of normal; creatinine of > 221 μmol/L); (3) uncorrectable bleeding or severe coagulopathy; (4) a target GSV diameter of < 3 mm or > 12 mm; (5) symptomatic peripheral artery disease; (6) deep vein thrombosis (DVT) or pulmonary embolism (PE) ; (7) acute systemic infectious diseases; (8) superficial thrombophlebitis; (9) undergone treatment of the GSV in the target limb; (10) comorbidities, such as cancer, liver disease, or cardiac insufficiency, which may pose challenges during the trial or significantly reduce life expectancy; (11) allergies to lidocaine or cyanoacrylates; or (12) non-primary varicose veins due to pelvic tumors, DVT, Klippel-Trenaunay syndrome, or arteriovenous fistula; (13) GSV was extremely distorted and thus unsuitable for minimally invasive treatment; (14) they were pregnant and/ or lactating; (15) they were participating in clinical trials of other drugs or medical devices; or (16) they were ineligible for enrollment due to other reasons.

### Procedure

The target vein and its most distal end were visualized under ultrasound guidance, and local anesthesia was administered at the selected intervention site. Under ultrasound guidance, the Seldinger technique was used to puncture the target vein and insert a support catheter. A 0.035-inch J-tip guidewire was advanced through the support catheter into the venous cavity toward the saphenofemoral junction (SFJ). Subsequently, a 7-Fr long catheter was introduced along the J-tip guidewire approximately 4–6 cm from the SFJ. The adhesive was loaded into a perfusion catheter and pushed to within 2–4 cm from the catheter tip. The long catheter was then retracted by 5 cm before the perfusion catheter was introduced until it was securely locked by the guide device. The perfusion catheter was determined to be 5 cm from the SFJ using ultrasound. The ultrasound probe was positioned 2–3 cm above the opening of the catheter to ensure complete SFJ closure. Simultaneously, the trigger was pulled for 3 seconds to distribute 0.1 mL adhesive into the vein, and the catheter was immediately pulled out by 1 cm. The trigger was pulled again to distribute a further 0.01 mL of adhesive and then pulled out by 3 cm. For every 3 cm of pull out, 0.1 mL adhesive was injected and the proximal end was compressed for 30 seconds until the target vein was completely closed. Finally, ultrasound was used to validate the treatment effect.

### Endpoints

The primary endpoint was the rate of complete GSV closure at 3 months. Complete closure was defined as a maximum open length of 5 cm in the treated vein, as determined by Doppler ultrasound. The secondary endpoints included the rate of complete GSV closure at 12 months, procedural duration, pain score, ecchymosis score, and Venous Clinical Severity Score (VCSS) and Aberdeen Varicose Vein Questionnaire Score (AVVQS) before and after surgery. To assess the safety of the device, adverse events were recorded.

### Statistical analysis

SAS software (version 9.4 or higher) was used for the statistical analyses. All tests were two-sided, and *P* < 0.05 was considered statistically significant. Quantitative indicators included the mean, standard deviation, median, minimum, maximum, lower quartile (Q1), and upper quartile (Q3). The t-test (assuming homogeneity of variance and a normal distribution) or the Wilcoxon rank-sum test was used to compare quantitative data between the groups. The χ^2^ test or Fisher’s test was used to compare categorical data. The Wilcoxon rank-sum test or the Cochran-Mantel-Haenszel test was used to compare ranked data.

### Non-inferiority margin

The primary efficacy results were compared between the groups using non-inferiority testing based on a one-tailed α of 0.025. The non-inferiority margin was determined to be -10%.

The expected closure rate was 95%, which is based on a study evaluating competing products (VenaSeal Closure System),^[4]^ and the effective closure rate remained at 95% when considering the sample size estimation for this study. In accordance with expert consensus and guidelines, the closure rate should be observed for all therapeutic indicators, and the complete saphenous vein closure rate ranges from 86.7% to 97.7% at 3 months after surgery according to the research results of the VenaSeal Closure System and relevant literature on radiofrequency therapy. ^[9, 10, 11, 12]^ The weighted average result of these literature findings was 95.35%, thus setting an expected closure rate of 95% for this study.

The non-inferiority margin was set at 10%. Determination of the non-inferiority margin followed a two-step approach. First, the absolute effect of the control device was estimated after accounting for comfort or relative effect (M1) through analysis. Second, the non-inferiority margin (M2) (M2 = f × M1) was determined, considering an appropriate ratio (1-f) that reflects specific clinical conditions alongside control device effects. Typically, f ranged from 0 to 0.5. For our main evaluation index (3-month rate of complete saphenous vein closure), M1 represented its actual clinical effect (preset as 95%) relative to the comfort or blank effect. The *F*-value was assigned as 0.1, resulting in a non-inferiority margin (M2) of 10%. Additionally, consideration was given to clinical recognition levels, where the researchers agreed that an acceptable threshold for non-inferiority was within a range of ± 10%.

## Results

**Figure 1:**
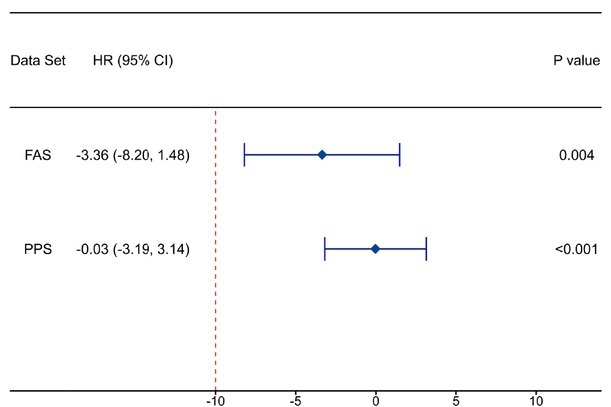
A total of 177 patients were enrolled and randomly allocated to either the GA group (*n* = 90) or the RFA group (*n* = 89). The SS included patients who received the trial device and underwent randomization based on the intention-to-treat principle. The FAS included patients who successfully completed the trial, excluding those with significant protocol violations. The PPS included patients who received the experimental device, underwent randomization, and underwent at least one safety assessment. FAS, full analysis set; PPS, per-protocol set; SS, safety set.

Overall, 177 patients were treated at nine centers, including 89 patients in the GA group and 88 patients in the RFA group. Four patients dropped out from the GA group due to loss to follow-up or other reasons, while one patient dropped out from the RFA group (Figure 1). The mean age of the patients was 56.25 ± 12.23 years, the mean body mass index was 24.34 ± 2.84 kg/m^2^, and 61.58% of the patients were female. No significant differences in baseline characteristics were observed between the two groups (Table 1).

**Table 1:** Characteristics of the patients and lesion severity

Characteristics	GA	RFA	Statistical magnitude	*P* value
Patients	*n* = 89	*n* = 88		
Age (years)*	56.85 ± 12.47	55.65 ± 12.03	-0.842 (Wilcoxon)	0.400
Sex ratio (M)	38 (42.70%)	30 (34.09%)	1.385 (χ^2^)	0.239
BMI (kg/m^2^)*	24.29 ± 3.12	24.39 ± 2.56	-0.242 (*t*-test)	0.809
Venous thrombus	0 (0%)	0 (0%)		
Diabetes	10 (11.24%)	10 (11.36%)	0.001 (χ^2^)	0.979
Hypertension	24 (26.97%)	26 (29.55%)	0.145 (χ^2^)	0.703
History of surgery	32 (35.96%)	39 (44.32%)	1.288 (χ^2^)	0.256
History of allergy	5 (5.62%)	7 (7.95%)	0.382 (χ^2^)	0.536
Legs	*n* = 89	*n* = 88		
One-side	76 (85.39%)	71 (80.68%)	0.698 (χ^2^)	0.404
Pain	39 (43.82%)	33 (37.50%)	0.732 (χ^2^)	0.392
GSV Diameter (mm)*	7.86 ± 2.05	8.02 ± 2.13	0.571 (Wilcoxon)	0.568
Lesion length (mm)*	27.64 ± 12.14	30.23 ± 10.53	1.890 (Wilcoxon)	0.059
Clinical class				
C0	0	0	0.000 (CMH)	0.995
C1	0	0		
C2	37 (41.57%)	35 (39.77%)		
C3	24 (26.97%)	26 (29.55%)		
C4_a_	26 (29.21%)	26 (29.55%)		
C4_b_	2 (2.25%)	1 (1.14%)		
C5	0	0		
C6	0	0		
VCSS*	3.55 ± 1.83	3.68 ± 1.66	0.668 (Wilcoxon)	0.504
AVVQS*	3.98 ± 2.60	4.88 ± 3.20	1.757 (Wilcoxon)	0.079

Values in parentheses are percentages, unless otherwise specified; *Values are mean ± standard deviation. The Wilcoxon rank-sum test was used for data analysis; the χ2 test was used for data analysis; the CMH test was used for data analysis. *P* < 0.05 was considered statistically significant. AVVQS, Aberdeen Varicose Vein Questionnaire Score; BMI, body mass index; CMH, Cochran–Mantel–Haenszel; GA, glue ablation; GCS, great saphenous vein; RFA, radiofrequency ablation; VCSS, Venous Clinical Severity Score.

### Primary outcome

Rate of complete GSV closure at 3 months after surgery. The rate of complete GSV closure at 3 months after surgery was satisfactory in both the GA and RFA groups. In the full analysis set (FAS), the rate of complete GSV closure was 95.51% in the GA group and 98.86% in the RFA group (*P* = 0.164). The difference between the GA group and the RFA group was -3.36% (95% confidence interval [CI] -8.20% to 11.48%, Pnon- inferiority = 0.004).

**Figure 2:**
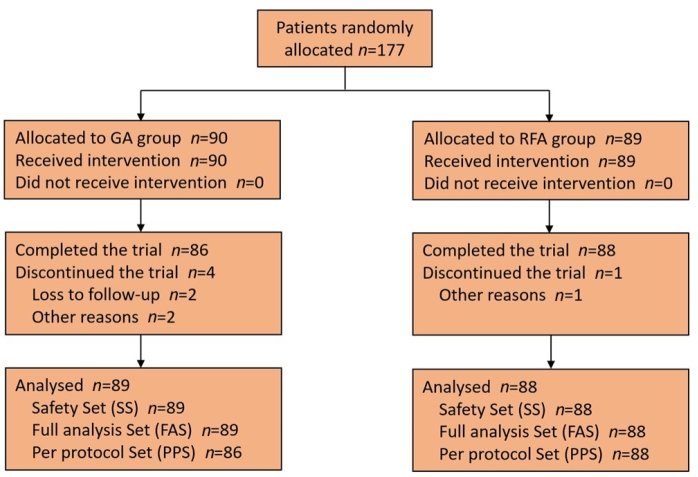
The disparity in the rate of 3-month complete GSV closure between the GA group and the RFA group. CI, confidence interval; FAS, full analysis set; GA, glue ablation; GSV, great saphenous vein; HR, hazard ratio; PPS, per-protocol set. P values indicate a test for interaction; *P* < 0.05 was considered statistically significant.

In the per-protocol set (PPS), the complete closure rate was 98.84% in the GA group and 98.86% in the RFA group (*P* = 0.894). The difference between the GA group and the RFA group was -0.03% (95% CI -3.19% to 3.14%, Pnon- inferiority < 0.001) (Table 2, Table 3). The 3-month rate of complete GSV closure in the GA group was deemed non-inferior to that in the RFA group as the lower boundary of the 95% CI for the between-group difference in the complete closure rate (in either the FAS or the PPS) exceeded the pre-defined noninferiority margin of -10% (Figure 2).

**Table 2:** Rate of complete GSV closure at 3 months after surgery

Parameters	FAS		PPS	
	GA	RFA	GA	RFA
Patients (missing)	89 (0)	88 (0)	86 (0)	88 (0)
Complete closure rate *n* (%)	85 (95.51%)	87 (98.86%)	85 (98.84%)	87 (98.86%)
Rate difference 95%CI (%)	-8.20, 1.48		-3.19, 3.14	
Statistical magnitude (CMH)	1.936		0.018	
*P* value	0.164		0.895	

The CMH-χ^2^ test was used for comparisons between groups. The difference in the rate of complete closure between groups was calculated as GA group – RFA group. The Worst Observation Carried Forward method was used to handle missing data in the FAS, which means the patients with missing endpoint results were considered as futile cases. CMH, Cochran–Mantel–Haenszel; FAS, full analysis set; GA, glue ablation; PPS, per-protocol set; RFA, radiofrequency ablation.

**Table 3:** Non-inferiority test of the rate of complete GSV closure at 3 months after surgery

Parameters	Data set	Statistical magnitude	*P* value	Rate difference 95%CI (%)
3-months complete	FAS	2.667	0.004	-3.36 (-8.20, 1.48)
Closure rate of GSV	PPS	6.171	<0.001	-0.03 (-3.19, 3.14)

Data from both the FAS and the PPS were tested for non-inferiority with regard to the difference in the rate of complete GSV closure at 3 months after surgery. *P* < 0.05 was considered statistically significant. The non-inferiority margin was –10%. CI, confidence interval; FAS, full analysis set; GSV, great saphenous vein; PPS, per-protocol set.

**Figure 3:**
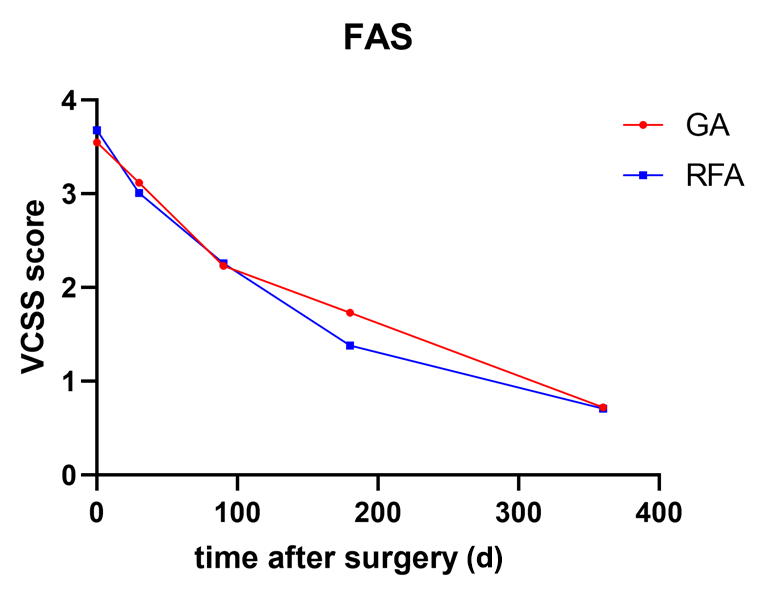
Trend in the VCSS in the GA and RFA groups within 12 months after surgery (FAS). FAS, full analysis set; GA, glue ablation; RFA, radiofrequency ablation; VCSS, Venous Clinical Severity Score.

**Figure 4:**
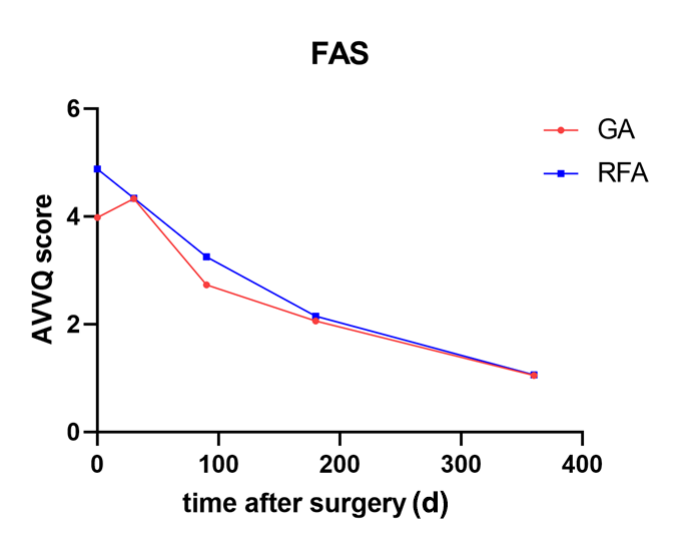
Trend in the AVVQS in the GA and RFA groups within 12 months after surgery (FAS). AVVQS, Aberdeen Varicose Vein Questionnaire Score; FAS, full analysis set; GA, glue ablation; RFS, radiofrequency ablation.

### Secondary outcome

Rate of complete GSV closure at 12 months after surgery. At 12 months, the GA group had 14 missing patients, while the RFA group had 17 missing patients. The dropout rate was not significantly different between the two groups (*P* = 0.425). The analysis of both the FAS and PPS indicated that the rate of complete GSV closure at 12 months in the GA group was significantly higher than that in the RFA group (100.00% *vs*. 92.96%; *P* = 0.023). The difference between the GA group and the RFA group was 7.04% (95% CI: 1.09% to 12.99%) (Table 4).

**Table 4:** Rate of complete GSV closure at 12 months after surgery

Parameters	FAS		PPS	
	GA	RFA	GA	RFA
Patients (missing)	75 (14)	71 (17)	75 (11)	71 (17)
Complete closure rate *n* (%)	75 (100.00%)	66 (92.96%)	75 (100.00%)	66 (92.96%)
Rate difference 95%CI (%)	1.09, 12.99		1.09, 12.99	
Statistical magnitude (CMH)	5.202		5.202	
*P* value	0.023		0.023	

The CMH-χ^2^ test was used for comparisons between groups. The difference in the rate of complete closure between groups was calculated as GA group – RFA group. *P* < 0.05 was considered statistically significant. CI, confidence interval; CMH, Cochran–Mantel–Haenszel; FAS, full analysis set; GA, glue ablation; GSV, great saphenous vein; PPS, per-protocol set; RFA, radiofrequency ablation.

### Procedural duration

In the FAS, 89 patients were included in the GA group, with a mean procedural duration of 22.34 ± 11.09 min and a median (min, max) procedural duration of 20 (8, 75) minutes. In the RFA group, there were 88 patients, with a mean procedural duration of 18.73 ± 9.82 min and a median (min, max) procedural duration of 16 (5, 46) minutes. The procedural duration was significantly longer in the GA group than in the RFA group in the FAS (*P* = 0.020).

The results obtained from the PPS were similar to those of the FAS. In the PPS, 86 patients were included in the GA group, with a mean surgical duration of 22.13 ± 11.13 min and a median (min, max) surgical duration of 20 (8, 75) minutes. The surgical duration in the RFA group was the same as in the FAS. Similar to the FAS, the surgical duration was significantly longer in the GA group than in the RFA in the PPS (*P* = 0.031) (Table 5).

**Table 5:** Comparison of total operative time among the randomized treatment groups

	FAS		PPS	
	GA	RFA	GA	RFA
Patients	89	88	86	88
Procedural time (min)*	22.34 ± 11.09	18.73 ± 9.82	22.13 ± 11.13	18.73 ± 9.82
Median (min)	20.00	16.50	20.00	16.50
Min, Max (min)	8.00, 75.00	5.00, 46.00	8.00, 75.00	5.00, 46.00
*P* value	0.020		0.031	

Values are presented as mean ± standard deviation, median, and minimum–maximum range. The Wilcoxon rank-sum test was used for comparisons between groups. *P* < 0.05 was considered statistically significant. FAS, full analysis set; GA, glue ablation; PPS, per-protocol set; RFA, radiofrequency ablation.

### Pain score

In the FAS, the mean immediate postoperative pain score was 1.36 ± 1.38 in the GA group and 1.42 ± 1.23 in the RFA group (Wilcoxon rank-sum *P* = 0.402). In the PPS, the immediate postoperative pain score was 1.34 ± 1.38 in the GA group and 1.42 ± 1.23 in the RFA group (Wilcoxon rank-sum *P* = 0.365) (Table 6).

**Table 6:** Pain intensity assessed using the Numeric Pain Rating Scale (NPRS,0-10) at 24 hours postoperatively

	FAS		PPS	
	GA	RFA	GA	RFA
Patients	89	88	86	88
Mean ± SD	1.36 ± 1.38	1.42 ± 1.23	1.34 ± 1.38	1.42 ± 1.23
Median	1.00	1.00	1.00	1.00
Min, Max (min)	0.00, 6.00	0.00, 5.00	0.00, 6.00	0.00, 5.00
*P* value	0.420	0.365		

The Wilcoxon rank-sum test was used for comparisons between groups. *P* < 0.05 was considered statistically significant. FAS, full analysis set; GA, glue ablation; PPS, per-protocol set; RFA, radiofrequency ablation.

**Table 7:** Assessment of postoperative ecchymosis severity among treatment groups FAS PPS

	FAS		PPS	
	GA	RFA	GA	RFA
Patients (missing)	88 (1)	85 (3)	85 (1)	85 (3)
Level 0: 0%, *n* (%)	33 (37.50%)	28 (32.94%)	33 (38.82%)	28 (32.94%)
Level 1: <25%, *n* (%)	49 (55.68%)	47 (55.29%)	47 (55.29%)	47 (55.29%)
Level 2: 25% and <<0%, *n* (%)	6 (6.82%)	9 (10.59%)	5 (5.88%)	9 (10.59%)
Level 3: 50% and <<5%, *n* (%)	0 (0.00%)	1 (1.18%)	0 (0.00%)	1 (1.18%)
Level 4: 75% and <<00%, *n* (%)	0 (0.00%)	0 (0.00%)	0 (0.00%)	0 (0.00%)
Level 5: 100%, *n* (%)	0 (0.00%)	0 (0.00%)	0 (0.00%)	0 (0.00%)
*P* value	0.267		0.181	

100% indicates that ecchymosis had exceeded the treatment area. The CMH test was used for data analysis. *P* < 0.05 was considered statistically significant. FAS, full analysis set; GA, glue ablation; PPS, per-protocol set; RFA, radiofrequency ablation.

### Ecchymosis score

One week after surgery, ecchymosis in the surgical area was assessed. The majority of patients had < 25% ecchymosis coverage in the surgical area, with one patient from the RFA group exhibiting > 50% coverage. The Cochran–Mantel–Haenszel test revealed no significant difference in ecchymosis between the GA and RFA groups.

### VCSS and AVVQS before and after surgery

The VCSS scores were analyzed in both groups within 12 months after surgery. In the FAS, both groups exhibited a consistent decline in the VCSS (Figure 1). The differences

between each timepoint and the baseline data were calculated. There was no significant difference in the VCSS between the GA group and the RFA group at 30 days (-0.43 ± 2.56 *vs*. -0.67 ± 2.41, respectively; *P* = 0.592), 3 months (-1.29 ± 2.58 *vs*. -1.42 ± 2.33, respectively; *P* = 0.570), 6 months (-1.81 ± 2.95 *vs*. –2.31 ± 2.14, respectively; *P* = 0.487), or 12 months (-2.75 ± 1.93 *vs*. -2.90 ± 1.83, *P* = 0.430) after surgery.

The AVVQS decreased progressively over time (Figure 2). The differences between each timepoint and the baseline data were calculated. There was no significant difference between the GA group and the RFA group at 30 days (0.35 ± 4.02 *vs*. -0.53 ± 4.30, respectively; *P* = 0.218), 3 months (-1.23 ± 3.31 *vs*. -1.63 ± 3.32, respectively; *P* = 0.216), or 6 months (-1.99 ± 3.31 *vs*. -2.73 ± 3.26, respectively; *P* = 0.112) after surgery. However, at 12 months after surgery, there was a significant difference between the GA group and the RFA group (-2.87 ± 3.01 *vs*. -3.83 ± 3.53, respectively; *P* = 0.031).

### Safety evaluation

The incidence of adverse events was documented within a 6–12-month postoperative period. In the GA group, there were 39 adverse events reported among 15 patients. In the RFA group, there were 25 adverse events in 14 patients. None of these adverse events were considered to be trial- or device-related.

## Discussion

GA is a novel non-thermal method of vein closure, which utilizes cyanoacrylate as its primary active ingredient. Cyanoacrylate, which is a liquid adhesive, rapidly polymerizes upon contact with anionic solutions like blood, resulting in vascular obstruction, inflammation, and fibrosis.^[13]^ In view of the potential concern that thermal ablation may cause injury, GA has gradually garnered attention due to its unique advantages. As such, it has emerged as a new approach for the minimally invasive treatment of varicose veins. The efficacy of this treatment for varicose veins has been demonstrated in several studies, with a GSV closure rate of > 90% over a 12-month follow-up period.^[14,15]^ Cyanoacrylate glue is considered safe, with low reported complication rates, including pain, thrombophlebitis, cellulitis, skin pigmentation, and deep vein thrombosis.^[16,17]^ The available evidence suggests that GA is anticipated to be a non-inferior alternative to RFA, exhibiting a lower incidence of postoperative complications. Nevertheless, further validation through large-scale clinical trials remains imperative.

The results of this 12-month randomized, parallel-controlled, multicenter, non-inferiority study demonstrate that GA is non-inferior to RFA in the rate of complete GSV closure at 3 months, with a closure rate of 95.51%, which is consistent with the findings of other contemporary studies. The rate of complete GSV closure with GA was 100% at 12 months, surpassing that of RFA (92.96%). Despite a dropout rate of 15.73%, potentially introducing bias to the results, GA still demonstrated satisfactory surgical efficacy. The VCSS and AVVQS also indicated that GA is comparable to RFA in terms of symptom improvement, and both methods achieved sustained clinical success. In terms of safety, it is worth affirming that there were no device-related adverse events with GA during the postoperative period of 6–12 months. Currently, the management of varicose veins is primarily focused on minimizing postoperative complications and optimizing perioperative quality of life. Foam sclerotherapy has gradually emerged as the predominant non-thermal ablation treatment due to its simplicity, speed, and minimal impact on quality of life. However, it is crucial not to overlook the primary objective of achieving reliable and durable elimination of venous reflux. Several studies have demonstrated that the 1-year anatomic success rate of foam sclerotherapy ranges between 70% and 75%,^[18,19]^ which is significantly lower than that with thermal ablation techniques.^[20]^ Consequently, there are reservations regarding foam sclerotherapy as a first-line treatment for large veins, particularly when the vein diameter is ≥ 6 mm, as this substantially increases the risk of treatment failure.^[21,22]^ Given the comparable efficacy of GA for treating GSV reflux, its gradual emergence as the primary non-thermal surgical approach can be anticipated.

Without thermal energy, GA has long been considered to be less painful and less invasive to the subcutaneous tissue than thermal ablation. However, in our study, there were no significant differences in the postoperative ecchymosis area and pain scores between the GA and RFA groups. This may be attributed to the fact that pain and ecchymosis primarily arise from treatment of the lower leg branch, rather than the method used to close the trunk of the GSV. The application of tumescent anesthesia in thermal ablation has been a significant advancement in surgical techniques in recent years.^[23]^ Previous studies have demonstrated that the use of tumescent anesthesia can effectively reduce surgical complications, such as skin burns and nerve damage. In the context of tumescent anesthesia, RFA can be successfully utilized for the treatment of large-diameter veins, particularly those > 12 mm in diameter, without compromising treatment outcomes.^[24]^ In our study, tumescent anesthesia was administered to all patients who underwent RFA, while it was not administered to patients in the GA group, potentially contributing to this finding.

We also conducted statistical analysis on the procedural duration, observing that GA exhibited a significantly longer surgical duration than RFA, which is contrary to our initial expectations. It is possible that chemical ablation necessitates lengthier intraoperative compression than thermal ablation, despite avoiding the need for tumescent anesthesia. Additionally, considering that the GA technique is not yet widely adopted, the surgeon’s proficiency may also contribute to the prolonged surgical duration.

### Limitations

This study has some limitations that should be considered. Firstly, the study was limited to one-year postoperative follow-up data, consequently lacking longer-term observational evidence. Secondly, extended follow-up durations were associated with declining patient compliance and elevated attrition rates, potentially introducing a dilution effect in the outcomes.

## Conclusion

The present study demonstrated that GA was non-inferior to RFA in the treatment of GSV varices over a follow-up period of 3 months, with no additional postoperative complications. GA was proven to be both safe and efficacious. However, further extensive clinical research with longer follow-up periods is required to better understand the long-term performance of GA.

## References

[1] Whing J, Nandhra S, Nesbitt C, Stansby G (2021). Interventions for great saphenous vein incompetence. Cochrane Database Syst Rev.

[2] Eberhardt RT, Raffetto JD (2014). Chronic venous insufficiency. Circulation.

[3] Labropoulos N, Leon M, Nicolaides AN, Giannoukas AD, Volteas N, Chan P (1994). Superficial venous insufficiency: correlation of anatomic extent of reflux with clinical symptoms and signs. J Vasc Surg.

[4] Carradice D, Mekako AI, Mazari FA, Samuel N, Hatfield J, Chetter IC (2011). Clinical and technical outcomes from a randomized clinical trial of endovenous laser ablation compared with conventional surgery for great saphenous varicose veins. Br J Surg.

[5] Carradice D, Mekako AI, Mazari FA, Samuel N, Hatfield J, Chetter IC (2011). Randomized clinical trial of endovenous laser ablation compared with conventional surgery for great saphenous varicose veins. Br J Surg.

[6] Navarro L, Min RJ, Boné C (2001). Endovenous laser: a new minimally invasive method of treatment for varicose veins--preliminary observations using an 810 nm diode laser. Dermatol Surg.

[7] Andercou O, Stancu B, Coman HF, Cucuruz B, Noppeney T, Marian D (2023). Radiofrequency Thermal Ablation for the Treatment of Chronic Insufficiency of the Saphenous Vein-A Comparative Retrospective Study. Int J Environ Res Public Health.

[8] Paravastu SC, Horne M, Dodd PD (2016). Endovenous ablation therapy (laser or radiofrequency) or foam sclerotherapy versus conventional surgical repair for short saphenous varicose veins. Cochrane Database Syst Rev.

[9] Beteli CB, Rossi FH, de Almeida BL, Izukawa NM, Onofre Rossi CB, Gabriel SA (2018). Prospective, double-blind, randomized controlled trial comparing electrocoagulation and radiofrequency in the treatment of patients with great saphenous vein insufficiency and lower limb varicose veins. J Vasc Surg Venous Lymphat Disord.

[10] Beyaz MO, Oztas DM, Ulukan MO, Arslan HM, Unal O, Ugurlucan M (2022). Preliminary Results of a New Illuminated Radiofrequency Ablation Catheter for the Treatment of Great Saphenous Vein Reflux Disease. Surg Innov.

[11] Morrison N, Gibson K, McEnroe S, Goldman M, King T, Weiss R (2015). Randomized trial comparing cyanoacrylate embolization and radiofrequency ablation for incompetent great saphenous veins (VeClose). J Vasc Surg.

[12] Nordon IM, Hinchliffe RJ, Brar R, Moxey P, Black SA, Thompson MM (2011). A prospective double-blind randomized controlled trial of radiofrequency versus laser treatment of the great saphenous vein in patients with varicose veins. Ann Surg.

[13] Linfante I, Wakhloo AK (2007). Brain aneurysms and arteriovenous malformations: advancements and emerging treatments in endovascular embolization. Stroke.

[14] Proebstle TM, Alm J, Dimitri S, Rasmussen L, Whiteley M, Lawson J (2015). The European multicenter cohort study on cyanoacrylate embolization of refluxing great saphenous veins. J Vasc Surg Venous Lymphat Disord.

[15] Bootun R, Lane TR, Davies AH (2016). The advent of non-thermal, non-tumescent techniques for treatment of varicose veins. Phlebology.

[16] Almeida JI, Javier JJ, Mackay E, Bautista C, Proebstle TM (2013). First human use of cyanoacrylate adhesive for treatment of saphenous vein incompetence. J Vasc Surg Venous Lymphat Disord.

[17] Tang TY, Rathnaweera HP, Kam JW, Chong TT, Choke EC, Tan YK (2019). Endovenous cyanoacrylate glue to treat varicose veins and chronic venous insufficiency-Experience gained from our first 100+ truncal venous ablations in a multi-ethnic Asian population using the Medtronic VenaSeal™ Closure System. Phlebology.

[18] Biemans AA, Kockaert M, Akkersdijk GP, van den Bos RR, de Maeseneer MG, Cuypers P (2013). Comparing endovenous laser ablation, foam sclerotherapy, and conventional surgery for great saphenous varicose veins. J Vasc Surg.

[19] Devereux N, Recke AL, Westermann L, Recke A, Kahle B (2014). Catheter-directed foam sclerotherapy of great saphenous veins in combination with pre-treatment reduction of the diameter employing the principals of perivenous tumescent local anesthesia. Eur J Vasc Endovasc Surg.

[20] Rasmussen LH, Lawaetz M, Bjoern L, Vennits B, Blemings A, Eklof B (2011). Randomized clinical trial comparing endovenous laser ablation, radiofrequency ablation, foam sclerotherapy and surgical stripping for great saphenous varicose veins. Br J Surg.

[21] Myers KA, Jolley D, Clough A, Kirwan J (2007). Outcome of ultrasound-guided sclerotherapy for varicose veins: medium-term results assessed by ultrasound surveillance. Eur J Vasc Endovasc Surg.

[22] Shadid N, Nelemans P, Lawson J, Sommer A (2015). Predictors of recurrence of great saphenous vein reflux following treatment with ultrasound-guided foamsclerotherapy. Phlebology.

[23] Merchant RF, Pichot O (2005). Closure Study Group. Long-term outcomes of endovenous radiofrequency obliteration of saphenous reflux as a treatment for superficial venous insufficiency. J Vasc Surg.

[24] Merchant RF, Pichot O, Myers KA (2005). Four-year follow-up on endovascular radiofrequency obliteration of great saphenous reflux. Dermatol Surg.

